# Supplementary material for: Phenotypes associated with genetic determinants of type I interferon regulation in the UK Biobank: a protocol
Source: Wellcome Open Res. 2023 Nov 23;8:550. [Version 1] doi: 10.12688/wellcomeopenres.20385.1 (PMC11162527; doi:10.12688/wellcomeopenres.20385.1)
Supplement: Peer review history

# Reviewer report: Reviewer response for version 1

**DOI:** 10.21956/wellcomeopenres.22572.r82014

**Published:** 2024-06-08

**Authors:** Guojun Hou

**Affiliations:** 1 Shanghai Institute of Rheumatology, Renji Hospital, Shanghai Jiao Tong University School of Medicine (SJTUSM), Shanghai, China

The authors outline the methodology for a hypothesis-driven investigation aimed at elucidating the genetic correlation between the interferon pathway and prevalent disorders. The study is well-justified, considering the robust mechanistic links established between IFN signaling and the molecular mechanisms underlying these diseases. This research serves as a model for examining the genetic connections between pathways pertinent to diseases and the diseases themselves.

Is the study design appropriate for the research question?

Yes

Is the rationale for, and objectives of, the study clearly described?

Yes

Are sufficient details of the methods provided to allow replication by others?

Yes

Are the datasets clearly presented in a useable and accessible format?

Yes

Reviewer Expertise:

My research focuses on utilizing gene editing to investigate the function of disease-associated genetic variants, however, I am not positioned to provide a competent evaluation of the statistical methods employed in this article.

I confirm that I have read this submission and believe that I have an appropriate level of expertise to confirm that it is of an acceptable scientific standard.

# Reviewer report: Reviewer response for version 1

**DOI:** 10.21956/wellcomeopenres.22572.r80683

**Published:** 2024-05-15

**Authors:** William A McEwan

**Affiliations:** 1 Institution: UK Dementia Research Institute, Cambridge, UK; 2 Department of Clinical Neurosciences, University of Cambridge, Cambridge, England, UK

Rioux et al describe the protocol of a proposed hypothesis-driven study to ascertain the genetic association between the interferon pathway and common neurological and cardiovascular diseases. The rationale for such a study is strong given that IFN signalling has been linked mechanistically to molecular processes underlying many diseases. Deeper understanding of this interplay gained by mining the UK Biobank would be highly valuable. 

 In neurodegeneration, there is strong observational and mechanistic evidence that the IFN system is important in disease, but currently a relative paucity of genetic association (absent a few genes downstream of IFN and LUBAC). It is not clear if this is because the IFN system is not a disease-driving process, or because the tools to identify risk variants lack power to identify rarer variants. This pathway-focussed study is therefore timely and important in this field.

 As many of the proposed phenotypes are potentially associated with infection, the control of which is an essential function of IFN, there is a possibility that genetic variants may be influencing infection risk or severity rather than the phenotype directly. Are there steps that could be included that would help expose the direct or indirect nature of the interaction?

 Parkinson's disease is not listed as a phenotype of interest in Supp Table 3 (other than in its contribution to all-cause dementia). Given recent evidence of a link between IFN and underlying molecular mechanisms (for example the ability of IFN-gamma to exacerbate LRRK2 variants (1)) and the appearance of IFN-regulated transcripts in disease (2) I would suggest PD as a strong candidate for inclusion in the study.

Is the study design appropriate for the research question?

Yes

Is the rationale for, and objectives of, the study clearly described?

Yes

Are sufficient details of the methods provided to allow replication by others?

Yes

Are the datasets clearly presented in a useable and accessible format?

Yes

Reviewer Expertise:

Molecular biology, protein aggregation. I am not a geneticist or clinician and cannot comment on the specifics of study design.

I confirm that I have read this submission and believe that I have an appropriate level of expertise to confirm that it is of an acceptable scientific standard.

## References

[1] : Interferon-γ signaling synergizes with LRRK2 in neurons and microglia derived from human induced pluripotent stem cells. *Nat Commun* .2020;11(1) : 10.1038/s41467-020-18755-4 5163 10.1038/s41467-020-18755-4 33057020 PMC7560616

[2] : Single cell analysis reveals the roles and regulatory mechanisms of type-I interferons in Parkinson's disease. *Cell Commun Signal* .2024;22(1) : 10.1186/s12964-024-01590-1 212 10.1186/s12964-024-01590-1 38566100 PMC10985960
